# Connecting the region during crisis: a Community of Practice response to the MV Hondius hantavirus outbreak

**DOI:** 10.5365/wpsar.2026.17.2.1488

**Published:** 2026-05-15

**Authors:** Jeyanthini Sathasivam, Wenyajing Zhang, Sarika Patel, Thilaka Chinnayah, Gina Samaan

**Affiliations:** a WHO Health Emergencies Programme, World Health Organization Regional Office for the Western Pacific, Manila, Philippines.; b Fellow of Epidemic Intelligence Programme, Ministry of Health, Putrajaya, Malaysia.

Just 10 days after a cluster of passengers with severe respiratory illness aboard a cruise ship was reported to the World Health Organization (WHO), (*1*) the Asia Pacific Health Security Action Framework Community of Practice (CoP) (*2*) mobilized to address emerging information and coordination needs during a fast-evolving public health event. Although the MV Hondius hantavirus outbreak did not primarily occur within the WHO Western Pacific Region, Member States recognized the potential implications of onboard contacts who had already been repatriated or were awaiting repatriation to their respective countries. In response to the urgent need for timely information-sharing and front-line preparedness, a 1-hour technical webinar was convened on 12 May 2026, drawing 2332 participants. The webinar presented a WHO situation update, along with Singapore’s experience and insights on quarantine-related activities. Importantly, the session served as a platform for rapid operational updates, allowing Member States and partners to learn, engage and communicate operational needs from countries and partners through the established CoP mechanism.

The webinar drew a diverse, multidisciplinary audience, reflecting broad regional and cross-sectoral engagement in hantavirus preparedness and response, as well as collective commitment towards strengthening regional readiness and response capacity. Attendees included epidemiologists, surveillance officers, clinicians, nurses, laboratory professionals, researchers and public health programme managers. Attendees also included professionals from One Health sectors, including animal health, environmental health and agriculture, as well as local government officers, community health workers, port health authorities, and communication and media personnel. Interest from regional and international public health networks, particularly within the Western Pacific Region, was clearly evident.

**Fig. 1:**
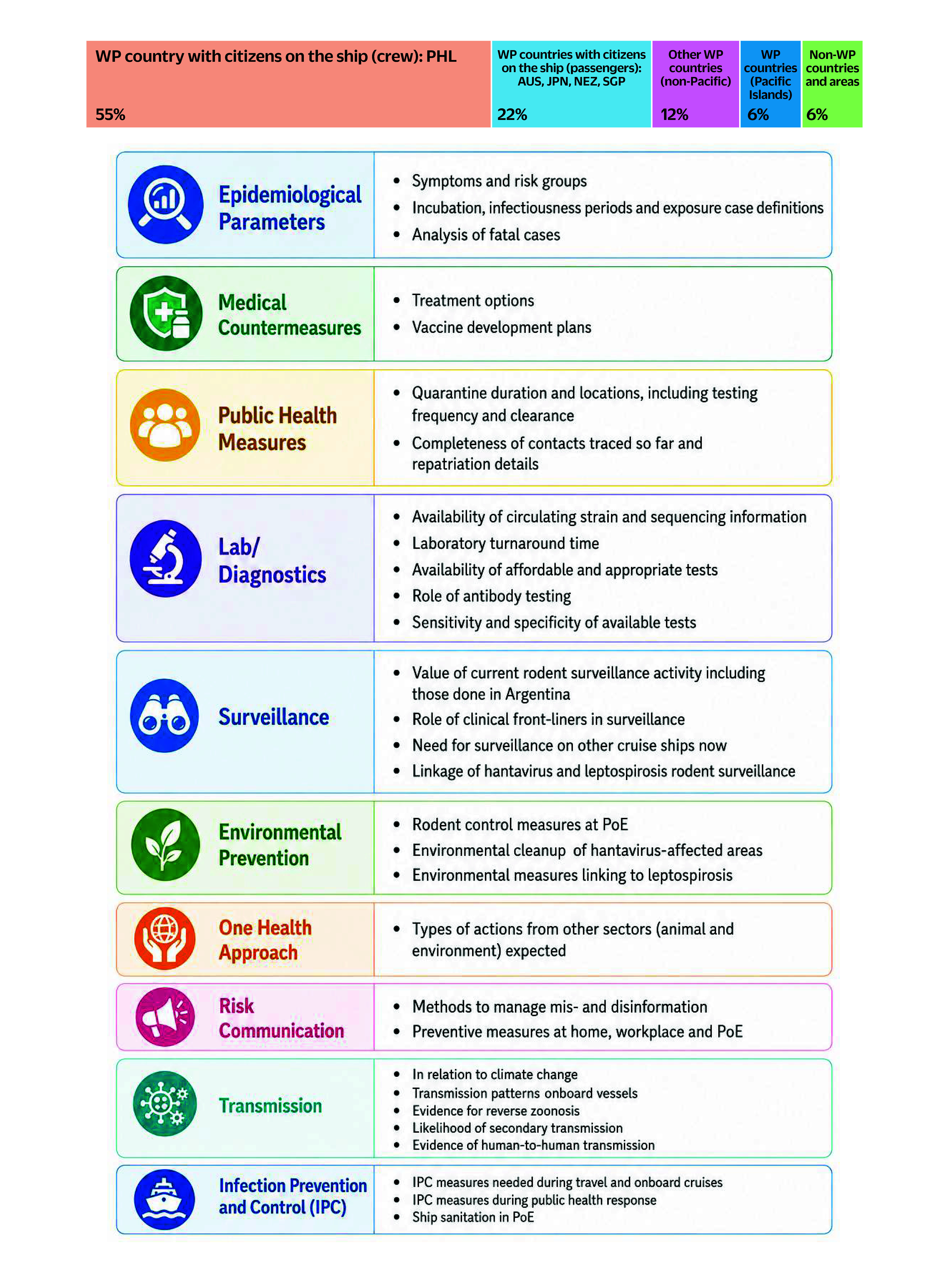
Participant representation (N = 2332) and questions raised during the webinar, 12 May 2026

Questions raised during the webinar reflected the wide-ranging concerns and information needs of participants as they respond to the evolving hantavirus event. Discussions focused on epidemiological parameters, including symptoms, incubation periods, infectiousness and analyses of fatal cases. Participants also sought information on medical countermeasures, including treatment options and vaccine development, as well as public health measures such as quarantine, contact tracing and repatriation procedures. Significant attention was given to laboratory diagnostics, surveillance systems, environmental prevention and One Health coordination. Concerns surrounding misinformation, transmission dynamics, climate-related influences, and infection prevention during travel and cruise operations further highlighted the complexity of preparedness and response efforts across sectors and countries (**Fig. 1**).

[**insert** Figure 1]

To gauge organizational preparedness, a rapid poll was conducted during the webinar. Of the 712 respondents, nearly half (49%) reported that their institutions were actively scaling up response activities, signalling heightened readiness across the region. The poll further revealed participants’ need for timely updates, technical guidance and continuous information-sharing, underscoring the importance of knowledge exchange and collaborative support in strengthening hantavirus preparedness and response.

The diversity of participants and the depth of engagement reflected substantial regional interest in hantavirus preparedness. This collective momentum underscores the critical importance of timely educational initiatives and sustained capacity-building to strengthen readiness for emerging infectious disease threats. By listening to and understanding Member States’ concerns, the region is better positioned to anticipate challenges, coordinate across sectors and respond efficiently to future emergent events, demonstrating that preparedness is not only a shared responsibility but also a cornerstone of resilience.
